# Cross-Sectional Study of Serum Vitamin B12 and Vitamin D3 Levels Amongst Corporate Employees

**DOI:** 10.7759/cureus.34642

**Published:** 2023-02-05

**Authors:** Virinchi Chirravuri, Swati Ghonge, Deepu Palal

**Affiliations:** 1 Community Medicine, Dr. D. Y. Patil Medical College, Hospital & Research Centre, Pune, IND

**Keywords:** deficiency, vitamin d3, vitamin b 12, vegetarians, corporate employees

## Abstract

Background

In today’s busy world, health is often neglected especially among full-time workers. Consequently, lifestyle disorders such as vitamin deficiencies are increasing, perhaps because of inadequate nutrition and lack of sunlight due to long hours working indoors. These deficiencies can lead to various short-term and long-term complications.

Objective

To estimate serum levels of vitamin B12 and vitamin D3 among vegetarian employees.

Methods and materials

A questionnaire about dietary and exercise habits was administered to participants who fulfilled the inclusion criteria and provided informed written consent. Participants also were asked about drug and supplement intake, history of smoking and alcohol, specific symptoms of vitamin B12 and D3 deficiency, and sociodemographic status. Blood samples were collected to estimate serum B12 and D3 levels.

Results

The results indicated that 14.00% of participants were vitamin B12 deficient and 82.00% were D3 deficient. Differences by gender were not statistically significant; vitamin B12 deficiency was identified in 10.00% of women and 14.44% of men, and vitamin D3 deficiency occurred in 100.00% of the women and 80.00% of men. Among 71 participants aged 35-45 years, 15.49% and 91.55% were deficient in B12 and D3, respectively; among 25 participants aged 46-55, 12.00% and 64.00% were deficient in B12 and D3, respectively; among four participants older than 55, no vitamin B12 deficiency was observed, but 25.00% were deficient in vitamin D3 (p=0.00002). Nearly all (96.15%) participants who reported never exercising were found to be vitamin D3 deficient, compared to 77.02% of those who exercised (OR=0.13, p=0.043). No significant association was found between alcohol consumption and vitamin B12 (p=1) or D3 (p=0.713) deficiency.

Conclusions

The results revealed a prevalence of both vitamin B12 and D3 deficiencies among corporate employees who identified as vegetarians. Increased awareness, dietary modifications, conscious physical activity, and most importantly, attention to one’s health may help improve vitamin sufficiency. Women over age 45 should pay particular attention due to their increased risk of vitamin D3 deficiency. Further research is needed to assess nutrition profiles among other populations to better understand vitamin deficiencies and design adequate preventive measures.

## Introduction

In today’s economy, many employees work long hours indoors. The lack of exposure to sunlight during office hours [1], in addition to an accompanying lack of exercise and nutrition, can have negative health impacts on employees, including decreased energy and stamina. Nutrition, particularly vitamin intake, provides essential micronutrients for proper functioning and metabolism [1]. Few studies have examined vitamin deficiencies among specific populations, such as vegetarians [2,3]. The aim of this study thus was to assess serum levels of vitamin B12 and D3 among vegetarian employees in India to create awareness and provide important data for policymakers and human resources management teams.

Vitamins can be classified as fat-soluble (e.g., vitamin D) or water-soluble (e.g., vitamin B12). Most vitamins are ingested with foods, and some are absorbed by the skin. For example, vitamin D3, or cholecalciferol, is synthesized in the skin during exposure to sunlight [4]. Vitamin D promotes calcium absorption in the gut and helps maintain adequate serum calcium and phosphate concentrations to enable normal bone mineralization, growth, and remodeling and to prevent hypocalcemic tetany, rickets, and osteomalacia [5-7]. Vitamin D deficiency may cause muscle pain in children and adults [8-10]. One study in people with alopecia areata showed that lower vitamin D blood levels were associated with more severe hair loss [11]. Vitamin D also modulates cell growth, neuromuscular and immune functions, and inflammation [6,12,13]; thus, a deficiency can lower one’s ability to fight infection [14,15]. Large observational studies have found a relationship between vitamin D deficiency and chronic lower back pain [16-18], as well as depression, particularly in older adults [19,20]. Together with calcium, vitamin D helps protect older adults from osteoporosis. Older adults are at increased risk of developing vitamin D deficiency because they cannot synthesize vitamin D as efficiently and are likely to spend long hours indoors [6]. Other risk factors for deficiency include working indoors and wearing protective clothing (e.g., long robes and head coverings, such as for religious reasons) [21,22]. Evidence indicates that rates of vitamin D3 deficiency have increased worldwide, even in tropical climates with abundant sunlight, such as urban India [23].

Vitamin B12 is required for central nervous system development and myelination, healthy red blood cell formation, and DNA synthesis and hemopoiesis [4,24-26]. The prevalence of vitamin B12 deficiency has increased. Symptoms of vitamin deficiency include weakness, fatigue, lightheadedness, heart palpations, shortness of breath, constipation, tingling sensations, and numbness. A study in Mumbai study showed that 65.00% of executives were at high risk for vitamin B12 deficiency, particularly vegetarians and the elderly [2].

## Materials and methods

This cross-sectional study was approved by the institutional ethics sub-committee (reference number I.E.S.C./40/2020) of Dr. D. Y. Patil Medical College, Hospital, and Research Centre in Pimpri, Pune, India. To identify eligible participants, a Google form questionnaire was circulated asking for the name, age, sex, occupation, and type of food consumed (vegetarian or non-vegetarian) by the respondent. Male and female employees working in the corporate sector and identified as vegetarians were included. Those who did not give consent, who identified as non-vegetarians, who reported taking vitamin supplements, or who were chronically and morbidly ill were excluded. Informed written consent was obtained after explaining the procedure in detail. Using a convenience sampling technique, the prevalence of Vitamin B12 and Vitamin D was 48.4% [27] and 67.9% [28], respectively. Considering a prevalence of 48.4%, within 95% confidence limit, and an absolute error of 10%, the minimum sample required was calculated to be 96. However, we included 100 study participants in our study. The software used was WinPepi, version 11.65 (Brixton Health, London, UK).

Each participant completed a detailed questionnaire containing information with respect to dietary and exercise patterns, drug and supplement intake, history of smoking and alcohol, symptoms of vitamin deficiency, and sociodemographic status. Using a Roche kit [29], blood samples were tested for serum B12 and D3 levels. In calculations for vitamin D3 (B12) levels, either deficiency or insufficiency (borderline deficiency) according to the reference range was considered as a deficiency, whereas sufficiency was considered normal. Specifically, vitamin D deficiency was defined as a serum level below 20 ng/mL, insufficiency as 20-30 ng/mL, and sufficiency as over 30 ng/mL. Vitamin B12 deficiency was defined as a concentration below 200 pg/ml, borderline deficiency as 200-300 pg/ml, and normal as over 300 pg/ml [30].

Data were analyzed using Microsoft Excel (Microsoft Corporation, Redmond, WA) and Epi Info software (CDC, Atlanta, GA). Descriptive statistics are presented as means and percentages. Tests of significance included the chi-square test for categorical data and the t-test.

## Results

**Figure 1:**
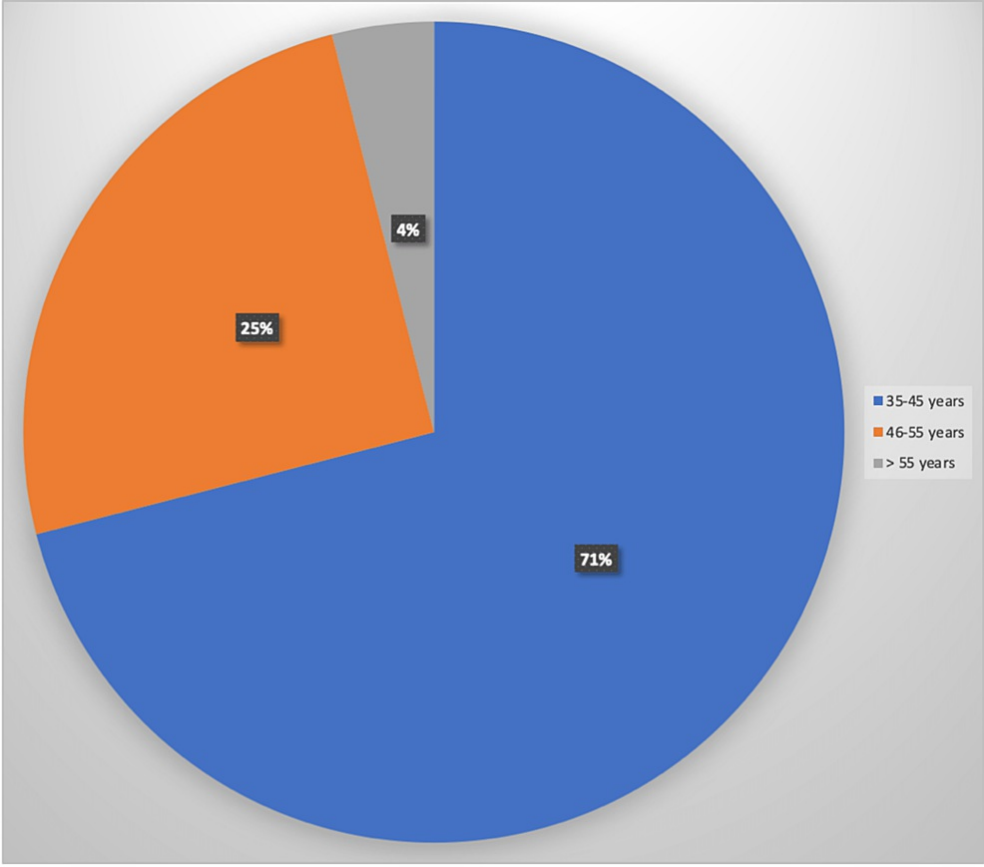
Percentage of participants in various age groups

Of the 100 corporate employees who took part in the study, 90 were males and 10 were females, with ages ranging from 35-45 (71.00%), 46-55 (25.00%), and 55-58 (4.00%) years (Figure 1). Most (86.00%) did not drink alcohol, 74.00% did some form of physical exercise, and 73.00% were COVID-free.

Comparing men and women, 10.00% of women and 14.44% of men were vitamin B12 deficient (Fisher exact P=1.00; odds ratio=0.6581; 95.00% confidence interval=0.0768-5.6385), and 100.00% of women and 80.00% of men were Vitamin D3 deficient (Fisher exact P=0.20) (Table 1).

**Table 1:** Prevalence of vitamin B12 and vitamin D3 deficiencies in the study population

	Vitamin B12			Vitamin D3		
	Deficiency (%)	Normal (%)	Total (%)	Deficiency (%)	Normal (%)	Total (%)
Women	1 (7.14%)	9 (10.47%)	10 (10.00%)	10 (12.20%)	0	10 (10.00%)
Men	13 (92.86%)	77 (89.53%)	90 (90.00%)	72 (87.80%)	18 (100.00%)	90 (90%)
Total	14 (100.00%)	86 (100.00%)	100 (100.00%)	82 (100.00%)	18 (100.00%)	100 (100.00%)
Special Test	Fisher exact P=1.000 Odds ratio=0.6581 95% confidence interval=0.0768-5.6385			Fisher exact P =0.2010		

**Figure 2:**
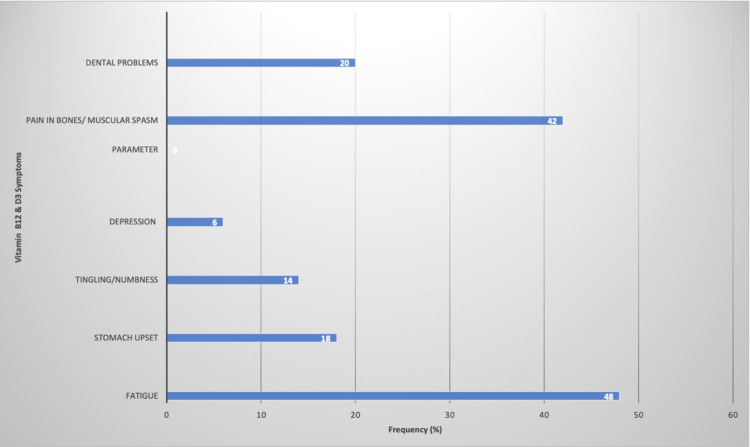
Percentage of participants with symptoms of vitamin B12 and D3 deficiencies

Regarding symptoms of D3 deficiency, 42.00% indicated bone pain or muscular spasms, and 20.00% reported dental problems. Regarding symptoms of vitamin B12 deficiency, 48.00% indicated experiencing fatigue, 18.00% had upset stomach, 14.00% had a tingling sensation or numbness, and 6.00% reported depression (Figure 2).

The blood test results indicated that 14.00% were vitamin B12 deficient and 82.00% were D3 deficient. Among the B12-deficient, the most common symptom was fatigue (48; 50.00%), among which 14.58% had serum levels indicating deficiency. Among those who reported upset stomachs (18; 21.43%), 16.67% had deficient serum levels. For those with tingling or numbness (14; 7.14%), 7.14% were actually deficient. Finally, among participants reporting depression (6; 6.00%), none were truly deficient (Table 2).

**Table 2:** Percentage of participants with symptoms versus those with actual B12 deficiency

	Fatigue			Stomach upset			Tingling or numbness			Depression		
	Present (%)	Absent (%)	Total (%)	Present (%)	Absent (%)	Total (%)	Present (%)	Absent (%)	Total (%)	Present (%)	Absent (%)	Total (%)
Deficiency	7 (14.58%)	7 (13.46%)	14 (14%)	3 (16.67%)	11 (13.41%)	14 (14%)	1 (7.14%)	13 (15.12%)	14 (14.00%)	0	14 (14.89%)	14 (14.00%)
Normal	41 (85.42%)	45 (86.54%)	86 (86%)	15 (83.33%)	71 (86.59%)	86 (86%)	13 (92.86%)	73 (84.88%)	86 (86.00%)	6 (100.00%)	80 (85.11%)	86 (86.00%)
Total	48 (100.00%)	52 (100.00%)	100 (100.00%)	18 (100.00%)	82 (100.00%)	100 (100.00%)	14 (100.00%)	86 (100.00%)	100 (100.00%)	6 (100.00%)	94 (100.00%)	100 (100.00%)
Tests	Chi-square P=1.00 Odds ratio=1.0976 95% confidence interval=0.3546-3.3973			Fisher exact P=0.713 Odds ratio=1.2909 95% confidence interval =0.32-5.19			Fisher exact P=0.685 Odds ratio=0.432 95% confidence interval =0.052-3.5909			Fisher exact P=0.5909		

Of those who were found deficient in vitamin D3, 42 (45.12%) indicated bone pain or muscle spasms, 88.1% of which had serum levels indicating deficiencies. Of the 20 (21.95%) who reported dental problems, 90.00% were found to be vitamin D3 deficient (Table 3).

**Table 3:** Percentage of participants with symptoms versus those with actual D3 deficiency

	Bone pain or muscle spasms			Dental problems		
	Present (%)	Absent (%)	Total (%)	Present (%)	Absent (%)	Total (%)
Deficiency	37 (88.10%)	45 (77.59%)	82 (82.00%)	18 (90.00%)	64 (80.00%)	82 (82.00%)
Sufficiency	5 (11.90%)	13 (22.41%)	18 (18.00%)	2 (10.00%)	16 (20.00%)	18 (18.00%)
Total	42 (100.00%)	58 (100.00%)	100 (100.00%)	20 (100.00%)	80 (100.00%)	100 (100.00%)
Special Test	Chi-square P=0.277 Odds ratio=2.1378 95% confidence interval=0.69-6.55			Fisher exact P=0.515 Odds ratio=2.25 95% confidence interval=0.4727-10.7099		

Of the 74 who said they exercise regularly, 12 (16.22%) and 57 (77.03%) were found deficient in vitamins B12 and D3, respectively. The mean level of vitamin B 12 was 550.92 pg/ml (U=948.5, Z=0.106, P=0.9155) in those who exercise regularly. The mean level of vitamin D3 was 23.04 ng/ml (U=679.5, Z=2.22, P=0.0264) (Table 4).

**Table 4:** Prevalence of vitamin deficiencies by exercise status

	Vitamin B12			Vitamin D3		
	Deficiency	Normal	Total	Deficiency	Normal	Total
Exercise	12 (85.71%)	62 (72.09%)	74 (74.00%)	57 (69.51%)	17 (94.44%)	74 (74.00%)
No exercise	2 (14.29%)	24 (27.91%)	26 (26.00%)	25 (30.49%)	1 (5.56%)	26 (26.00%)
Total	14 (100.00%)	86 (100.00%)	100 (100.00%)	82 (100.00%)	18 (100.00%)	100 (100.00%)
Special Tests	Fisher exact P=0.346 Odds ratio=2.3226 95% confidence interval=0.4835-11.1569			Fisher exact P=0.0362 Odds ratio=0.1341 95% confidence interval=0.0169-1.0638		

Of the 14 who reported consuming alcohol, 14.29% and 78.57% were found deficient in vitamins B12 and D3, respectively (Table 5).

**Table 5:** Prevalence of vitamin deficiencies by alcohol consumption

	Vitamin B12			Vitamin D3		
	Deficiency	Normal	Total	Deficiency	Normal	Total
Alcohol	2 (14.29%)	12 (13.95%)	14 (14.00%)	11 (13.41%)	3 (16.67%)	14 (14.00%)
No alcohol	12 (85.71%)	74 (86.05%)	86 (86.00%)	71 (86.59%)	15 (83.33%)	86 (86.00%)
Total	14 (100.00%)	86 (100.00%)	100 (100.00%)	82 (100.00%)	18 (100.00%)	100 (100.00%)
Special Tests	Fisher exact P=1.00 Odds ratio=1.03 95% confidence interval=0.204-5.1752			Fisher exact P=0.713 Odds ratio=0.77 95% confidence interval=0.1924-3.1189		

Finally, deficiency rates by age group were as follows: 15.49% and 91.55% were deficient in B12 and D3, respectively, among those aged 35-45; 12.00% and 64.00% among those aged 46-55; and 0% and 25.00% among the four participants older than 55 years (Table 6).

**Table 6:** Prevalence of vitamin deficiencies by age group

	Vitamin B12				Vitamin D3			
	Deficiency (%)	Normal (%)	Total (%)	Deficiency (%)		Normal (%)	Total (%)	
35–45 years	11 (78.57%)	60 (69.77%)	71 (71.00%)	65 (79.27%)		6 (33.33%)	71 (71.00%)	
46–55 years	3 (21.43%)	22 (25.58%)	25 (25.00%)	16 (19.51%)		9 (50.00%)	25 (25.00%)	
>55 years	0	4 (4.65%)	4 (4.00%)	1 (1.22%)		3 (16.67.00%)	4 (4.00%)	
Total	14 (100.00%)	86 (100.00%)	100 (100.00%)	82 (100.00%)		18 (100.00%)	100 (100.00%)	
Special Test	Fisher exact P=1.00			Fisher exact P=0.0001				

## Discussion

Previous studies conducted in 2007 and 2018 that included both vegetarian and non-vegetarian subjects showed that 60.00%-70.00% of vegetarians were deficient in B12 [2,3]. In contrast, the current study found that among 100 vegetarians, only 14.00% were deficient in B12, probably due to a conscious dietary plan rich in Vitamin B12. In the 2007 study, more than half (55.81%) of subjects with deficiency consumed alcohol, compared to 30.18% in the 2018 study [2,3]. In contrast, the current study results showed that only 14.00% of the 14 alcohol drinkers had a serum level indicating vitamin B12 deficiency. The lower rate of alcohol consumption in the current study, along with increased awareness and lifestyle modifications, could be the driving factors for better nutrition profiles.

With respect to physical exercise, the results of the current study corroborate those of the 2007 study [2], which showed that all non-exercisers had a vitamin B12 deficiency. In the current study, most (84.00%) of those who reported exercising were found to have normal B12 values. The results suggest a correlation between physical exercise and maintaining adequate vitamin B12 levels.

As for exercise and vitamin D3 deficiency, nearly all (96.15%) participants in the current study who exercised < 4 hours were found deficient, which is similar to the findings from the 2007 study [2]. However, it is interesting to note that most (77.00%) of those who reported exercising were also found deficient in vitamin D3. The high rates of deficiency could be due to lack of sun exposure or exercising indoors or in the evenings.

Similar to the 2007 study, which found that nearly half (48.00%) of the participants with leg pain had D3 deficiency [2], 45.12% of subjects in the current study who indicated experiencing bone pain were found to be deficient in D3. The result suggests a link between vitamin D3 and bone health. Although no difference in vitamin B12 deficiency was observed between men and women, all female subjects in our study were deficient in D3, compared to 80.00% of male subjects. A possible explanation for this overall deficiency is that modern work and family obligations severely limit individuals' exposure to sunlight. Vitamin deficiencies also were higher in younger (35-45 years) rather than older (>55 years) subjects.

## Conclusions

Work and lifestyle habits can significantly impact public and individual health. In the current study, corporate vegetarian employees were overall deficient in vitamins B12 and D3. However, participants over the age of 55 generally had better vitamin sufficiency levels, suggesting that younger people with sedentary jobs may not be getting adequate exercise and nutrition. Increased awareness of balanced diets and conscious physical activity may help improve these levels, especially among younger employees.

Overall, building awareness of the root causes, consciously identifying the opportunities to improve, and intentionally adopting healthy lifestyle management practices will help in alleviating Vitamin B12 and D3 deficiencies. It is also important to treat these deficiencies promptly to avoid long-term complications. The results reported here can be useful for policymakers and human resources professionals who want to educate employees about these conditions, including warning signs of nutritional deficiency.
